# Electroporation with Cisplatin against Metastatic Pancreatic Cancer:* In Vitro* Study on Human Primary Cell Culture

**DOI:** 10.1155/2018/7364539

**Published:** 2018-03-19

**Authors:** Olga Michel, Julita Kulbacka, Jolanta Saczko, Justyna Mączyńska, Piotr Błasiak, Joanna Rossowska, Adam Rzechonek

**Affiliations:** ^1^Department of Medical Biochemistry, Wroclaw Medical University, Wroclaw, Poland; ^2^Department of Molecular and Cellular Biology, Wroclaw Medical University, Wroclaw, Poland; ^3^Department of Thoracic Surgery, Wroclaw Medical University, Wroclaw, Poland; ^4^Institute of Immunology and Experimental Therapy, Polish Academy of Sciences, Wroclaw, Poland

## Abstract

Despite the rapid progression of cancer pharmacotherapy, the high drug resistance of pancreatic ductal adenocarcinoma (PDA) makes it one of the most lethal malignancies. Therefore, there are high expectations associated with experimental therapies, such as electrochemotherapy (ECT). This technique involves the application of short electric pulses to induce transitional permeabilization of the cellular membrane, thus enhancing drug molecules influx. The aim of the study was to investigate the influence of electroporation with cisplatin (CisEP) on the primary culture of human PDA cells from lung metastases—their survival and stress response. Considering the growing importance of various research models, two established human PDA cell lines, EPP85-181P (sensitive to daunorubicin) and EPP85-181RDB (resistant to daunorubicin), were utilized as a reference control. Cisplatin revealed higher cytotoxicity towards established cell lines. Following CisEP application, we observed a significant decrease of cells viability in the primary culture model. After CisEP therapy, an increased immunoreactivity with SOD-2 and Casp-3 antibodies was noticed. In conclusion, we discovered that electroporation can enhance the cytotoxic effect of cisplatin in pancreatic cancer cells* in vitro*. This effect was evident for cells from the primary culture. The obtained results confirm the importance of primary cells models in studies on the efficacy of experimental cancer therapies.

## 1. Introduction

Worldwide, over 200,000 people are diagnosed with pancreatic cancer every year. The highest risk of morbidity (approximately 8–12/10^5^ males and 6-7/10^5^ women) relates to the population of industrialized countries, where pancreatic cancer is the fifth most common cause of cancer-related mortality. In the United States, malignant tumors of the pancreas are currently one of the four leading causes of cancer-related death and are expected to become the second within the next two decades [1]. Such high mortality rates result from late diagnosis and extremely aggressive character of the neoplasm. No more than 25% of patients qualify for surgical intervention and, even after resection, a median survival amounts only 12 to 20 months [2]. Furthermore, at the time of diagnosis, roughly 50% of patients are burdened with metastasis. The survival rate for those patients does not exceed 6% [3]. Low treatment efficacy in conjunction with a number of side effects associated with chemotherapy causes a need for enhancement of classical cytostatics' activity or the evaluation of new drugs. Among methods supporting the transport of chemotherapeutic agents into cells, the electroporation (EP) seems to be promising. The basis of this technique is exposing the cell to the pulsed electric field action which can cause a dual effect:

- High voltage intensity application (1500–3000 V/cm) can lead to the so-called “total permeabilization” and consequently cell lysis [4, 5]. EP applied in this way is called an irreversible electroporation (IRE). While typical IRE protocols assume the delivery of a series of 50–100 *μ*s pulses, high-frequency IRE (H-FIRE) pulses require a higher energy dose to create equivalent lesions to standard IRE [6].
- The use of low-intensity electric field (<1500 V/cm) results in transient permeabilization of the cell membrane. Although numerous studies have demonstrated increased transportation of drugs such as bleomycin and cisplatin [7–9], the real cause of this phenomenon remains controversial. Whereas classic approach assumes the formation of hydrophilic pores in a structure of the cellular membrane, another theory reasons that the increase in the transmembrane voltage results in a disruption of the lipid bilayer enabling the entrance of water molecules “carrying” hydrophilic compounds [10, 11].

It was shown that the toxicity of cisplatin can be increased 3 to 13 times via electroporation, whereas electroporation with bleomycin decreases the viability of various cancer cells by a factor of 100 to several 100-fold [7, 12]. Due to the high efficacy of bleomycin, it is used as a first-choice drug for electrochemotherapy. Ongoing phase I/II studies of ECT on pancreatic cancer conducted at the National Cancer Institute “G. Pascale Foundation” of Naples did not show any serious side effects of electrochemotherapy in patients with locally advanced disease [13]. These results were confirmed by Granata et al. (2015) who also reported a reduced diameter and tumorigenicity of the lesions for a significant number of patients [14]. Remarkably, the only medicine used in the electrochemotherapy of patients with pancreatic cancer is bleomycin, which has been shown to cause alveolar cell damage and subsequent pulmonary inflammation [15]. One of the biggest advantages of ECT is the limitation of drug dosage and reduction of side effects. However, ECT is mostly performed on patients with cutaneous and subcutaneous tumors, and there is not adequate number of studies that can prove that there is no significant toxicity in patients with pancreatic cancer. Although toxicity incidence dramatically rises in patients receiving a dose in excess of 450 mg/m^2^, toxicity has been reported at doses of less than 100 mg and general anaesthesia following the use of bleomycin may be complicated by postoperative respiratory failure possibly secondary to a bleomycin-induced sensitivity of oxygen [16]. Since lung, liver, and peritoneum are the most common sites of metastasis [17], there is a need to implement alternative drugs for patients suffering from metastatic lung cancer and bleomycin-sensitive patients. In the presented study we performed electroporation with cisplatin, which is another drug officially approved for electrochemotherapeutic protocols.

Primary cell cultures are an integral part of modern biotechnology research. Compared to established cell lines, they are characterized by a much more reliable response to stress. Moreover, experiments carried out on cells taken from patients' tissues allow for the personalization of therapy, highly important in the treatment of various cancers. Higher reliability of such models is mainly attributed to the lower risk of disturbances in signaling and differentiation processes, which is characteristic for the established continuous cell cultures [18]. Unfortunately, the results obtained in this type of research are often ambiguous as every primary cell culture should be treated as a separate subpopulation. This is probably the main reason why they are reluctantly chosen cancer models. However, in the era of personalized medicine, establishing cultures for the targeted therapy should become standard. Here, we decided to perform the experiment on the primary cell culture derived from the pulmonary metastasis from pancreatic ductal adenocarcinoma. We hypothesized that electroporation enhances the efficacy of cisplatin in human pancreatic cancer cells from the primary cell culture.

## 2. Material and Methods

### 2.1. Cell Culture

**Figure 1:**
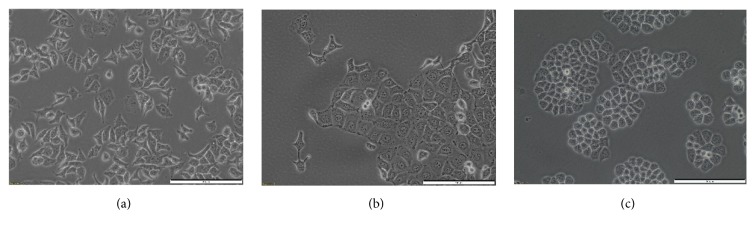
The morphology of the primary cell culture from pulmonary metastases of pancreatic cancer (a) and derived cell lines of pancreatic ductal adenocarcinoma sensitive to daunorubicin (EPP85-181P (b)) and resistant to daunorubicin (EPP85-181 RDB (c)).

The study was carried out* in vitro* on three models: two established cell lines EPP85-181P (sensitive to daunorubicin) and EPP85-181RDB (resistant to daunorubicin) and cells derived from pulmonary metastasis of pancreatic cancer. Both established cell lines were obtained from Institute of Pathology, University Hospital Charité in Berlin. Using defined cell lines with different mechanisms of drug resistance would enable us to initially classify the sensitivity of the primary cells to the pulsed electric field. In a further perspective, the obtained results may provide a link between the response to the ECT and the overexpression of different proteins responsible for the acquisition of drug resistance. Primary and fresh tumor samples were retrieved from a patient during surgery. The patient underwent a right-side videothoracoscopy under general anaesthesia. A biopsy of the pleural lesions was performed and the material for histopathological examination was obtained. At the same time, a part of the tumor was suspended in the culture medium. The postoperative course was without complications. Tumor material was processed directly after surgery. The cells were isolated from tissue fragment according to the procedure described previously [19]. Briefly, upon the arrival at the laboratory, the tissue was gently rinsed from blood cells with a sterile PBS buffer. Next, the collected samples were shredded with a scalpel in Petri dishes (Shutterstock, US) and suspended in dedicated culture medium. Part of the suspended material was immediately transferred on 75 cm^2^ culture flasks. For the first 3 days the medium was replaced daily, however, carefully not to discard not-attached fragments. Then, the medium was fully replaced twice a week. The average time to obtain confluence in both Petri dish and culture flask was approximately 14 days. Cells were cultured in modified high-glucose Leibovitz's L-15 medium (Gibco, Life Technologies, Carlsbad, CA) supplemented with 10% fetal bovine serum and 1% antibiotics (penicillin and streptomycin), 1.5% sodium bicarbonate (7.5%, Gibco), 1% MEM vitamin solution (Sigma, Saint Louis, MO), 0.5% ultraglutamine 1 (Lonza, Basel, Switzerland), 0.1% glucose (45%, Sigma), and 0.7% aprotinin (BioShop, Canada). Cultures were maintained at 37°C in a humidified, 5% carbon dioxide atmosphere. For experiments, we used fresh cells as well as the ones preserved in liquid nitrogen, collected from early passages (3 to 12). We compared the morphology of the primary cell culture with the continuous PDA cell lines of different degrees of drug resistance: EPP85-181P (sensitive to daunorubicin) and EPP85-181RDB (resistant to daunorubicin, overexpressing P-glycoprotein) (Figure 1).

Pancreatic adenocarcinoma origin of the primary cell culture was confirmed by histological analysis (Table 1). The distinguishing between pulmonary adenocarcinoma and fibroblasts was made according to literature [20] and the diagnostic procedures applied in clinical unit from where the tissue sections were collected; we examined the immunoreactivity of thyroid transcription factor 1 (TTF-1) mouse monoclonal antibody (Life Technologies, cat. no. 80221) in dilution 1 : 50, cytokeratin 7 (CK 7) mouse monoclonal antibody (Thermo Fisher Scientific, Waltham, MA; cat. no. MA1-06316) in dilution 1 : 100, and cytokeratin 20 (CK 20) mouse monoclonal antibody (Thermo Fisher Scientific, Invitrogen, cat. no. MA5-13263) in dilution 1 : 50. Additionally, we investigated the presence of immunocytochemical reaction with the pancreas-specific marker glycoprotein 2 (GP2) zymogen granule membrane mouse monoclonal antibody (Abcam, United States, cat. no. ab218410) in dilution 1 : 150.

The research was conducted in accordance with the requirements of the Bioethics Commission of Wroclaw Medical University.

### 2.2. Chemosensitivity Tests

cis-Diamminedichloridoplatinum(II) is a phase-specific chemotherapeutic drug, which fulfills its antitumor role primarily through the formation of cross-links between adjacent DNA strands and within the same strand, therefore limiting the DNA replication. In the first stage of the study, we assessed the toxicity of cisplatin towards the primary cell line and two continuous cell lines of PDA: EPP85-181P and EPP85-181RDB. For that purpose, cells were seeded at 8 × 10^3^ cells per well in 96-well plates, allowed to attach for 24 h and subsequently exposed to drugs for 24 or 72 hours to assess short- and long-term influence of different concentrations to cell viability. The drug was purchased from Sigma. Prior to use, the drug was dissolved in sterile PBS (Sigma) to 5 mM concentration and subsequently diluted with culturing medium to concentrations at a range of 0–50 *μ*M. The viability of the cells following treatment was determined by MTT (3-(4,5-dimethylthiazol-2-yl)-2,5-diphenyltetrazolium bromide, Sigma) test. MTT assay is a colorimetric method that estimates the rate of metabolism in viable cells. The absorbance was measured with multiwell scanning spectrophotometer at 570 nm (EnSpire Multimode Plate Reader, Perkin Elmer, Waltham, Massachusetts, USA). Cell viability was expressed as a percentage of treated cells compared to control cells, cultured in 96-well plates in medium for 24 and 72 hours.

### 2.3. Electroporation Protocol

Following harvesting, the cells were diluted in sterile EP buffer with low electrical conductivity (pH 7.4; 10 mM phosphate, 1 mM MgCl_2_, 250 mM sucrose), without drug presence, and the suspension of cells in buffer was electroporated in 4 mm cuvettes with parallel electrodes. Electroporation parameters were as follows: 8 pulses with time interval 100 *μ*s with 0–1400 V/cm range and 1 Hz using a BTX 830 square wave electroporator (BTX Harvard Apparatus, Syngen Biotech, Wroclaw, Poland). After electroporation, cells were incubated at 37°C for 10 minutes and subsequently centrifuged, diluted with culture medium, and seeded on 96-well microtiter plates. Parameters were optimized for high permeabilization and low cellular mortality by electroporation alone via MTT assay and flow cytometry with propidium iodide. Flow cytometry analysis was performed for assessment of the cell ability to internalize propidium iodide into the pancreatic cancer cells. Cells were detached with Trypsin-EDTA and subjected to EP protocol described above. Immediately before EP, propidium iodide (PI, P4170, Sigma) was applied to the cell suspension. The concentration of PI in cuvette for the EP buffer was 10 *μ*M. Then, the cells were incubated for 10 min at 37°C in a humidified atmosphere containing 5% CO_2_. After this time cells were washed in PBS and resuspended in 0.5 ml of PBS. Flow cytometric analysis was performed on a FACS Calibur flow cytometer (Becton Dickinson). The fluorescence of PI was measured with FL-3 detector. At least 10,000 viable cells were measured from each sample at a rate of up to 1000 cells/s.

### 2.4. Electrochemotherapy* In Vitro* Protocol

Cells were harvested and diluted in sterile EP buffer with 0, 5, or 10 *μ*M concentration of cisplatin. Next, they were electroporated and seeded on microtiter plates according to the procedure described in previous paragraph. The mitochondrial activity was measured 24, 48, and 72 hours after electroporation. Additionally, to assess the direct influence of the experiment, trypan blue staining was performed 30 min after electroporation and the percentage of living cells was determined using Bio-Rad TC20™ automated cell counter.

### 2.5. Immunocytochemical Staining

Immunocytochemistry was performed on treated cells fixed in 4% fresh paraformaldehyde 24 hours after treatment. Cells were stained using rabbit polyclonal anti-SOD2 antibody (Thermo Fisher Scientific, cat. no. LF-MA0030) and mouse monoclonal antibody against caspase 3, raised against amino acids 1-277 representing the full-length precursor form of human caspase-3 (Santa Cruz Biotechnology, Dallas, TX; cat. no. sc-7272). SOD2 is a mitochondrial manganese enzyme, which is the main scavenger of superoxide anions produced during the mitochondrial oxidative phosphorylation. The increased immunoreactivity may indicate the occurrence of oxidative stress after the applied therapy. Caspase 3 is an aspartate-specific cysteine protease which may be involved in extrinsic and intrinsic apoptotic pathways via caspase-dependent oligonucleosomal DNA fragmentation [21]. The increased immunoreactivity of casp-3 marker would be a first signal that apoptosis may occur in a particular cell line. Both antibodies were diluted with PBS buffer in a proportion 1 : 200. Briefly, after overnight incubation, the antigens were visualized with EXPOSE Specific HRP/DAB Detection IHC Kit (Abcam, Cambridge, UK) in compliance with the manufacturer's instructions. All probes were counterstained with Mayer's hematoxylin (Sigma). Samples after immunostaining were blinded for analysis. The stained cells were examined with the upright microscope (Olympus BX51, Hamburg, Germany) and expression was determined semiquantitatively as a percentage of positive cells stained (from a total of 150 cells per sample). The intensity of immunocytochemical staining was evaluated as (−) negative, (+) weak, (++) moderate, and (+++) strong.

### 2.6. Statistical Analysis

Three experimental replicates were performed for each* in vitro *experiment. The data were presented as the mean ± SD. For the electrochemotherapy experiments as there were two factors influencing viability (drug and electric pulses), we performed two-way ANOVA after the examination of the normal distribution. Cells subjected to both electroporation and cisplatin were compared to nontreated control, electroporation alone as well as with the particular parameter of cisplatin for the appropriate drug concentration. Other experiments were analysed via Student's *t*-test where following samples were compared to nontreated control. The differences were evaluated only within one time of incubation. Statistically significant differences between treated samples and controls were placed on graphs with *p* value of ^*∗*^*p* ≤ 0.05 being considered as significant. All statistical calculations were performed and analysed using Microsoft Excel, GraphPad Prism 7.0, and Statistica 13.1 software.

## 3. Results

### 3.1. Optimization of Drug Concentration

**Figure 2:**
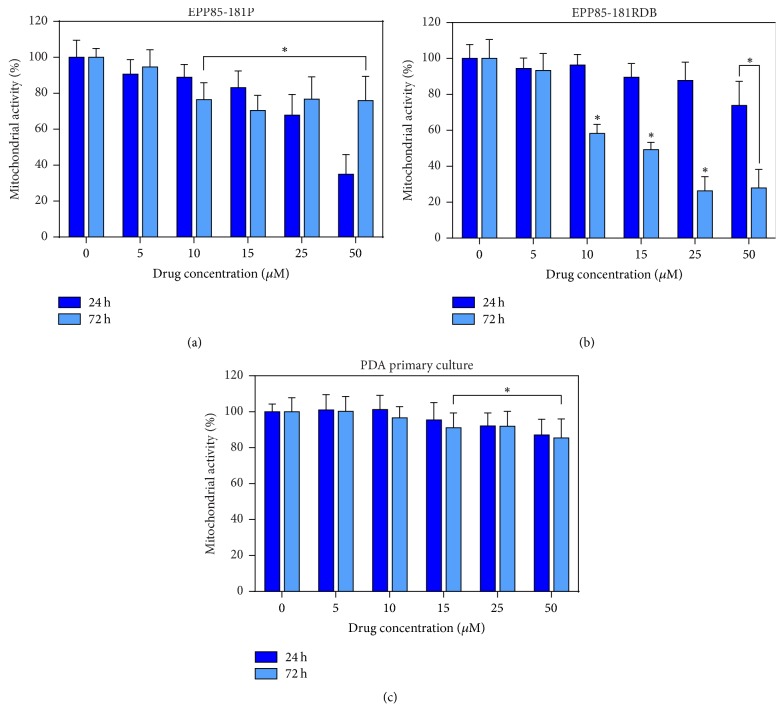
The results of MTT assay following cytotoxicity tests with cisplatin (Cis) at a range of 0–50 *μ*M concentration for 24 and 72 hours on established cell lines of PDA sensitive (EPP85-181P; (a)) and resistant to daunorubicin (EPP85-181RDB; (b)) and on the primary cell culture from the PDA metastasis (c); ^*∗*^statistically significant differences between cisplatin treated cells and nontreated control (*p* ≤ 0.05).

It has been shown that pancreatic cancer cells from primary cell culture are characterized by low sensitivity to cisplatin (Figure 2). Both cell lines, the sensitive one to daunorubicin and the resistant one, showed greater sensitivity to cisplatin when compared to the primary cells. Interestingly, notwithstanding the initial drop-in mitochondrial activity after 24 hours of incubation, the viability of the sensitive cell was not strongly affected as the subsequent increase was visible after 72 hours of incubation. On the contrary, despite the insufficient response of the resistant cell line, the later plummet suggests that, in fact, this cell line can be more sensitive to cisplatin than EPP85-181P. Two low-toxic concentrations of 5 and 10 *μ*M were selected for further experiments.

### 3.2. Optimization of the Electric Field Strength

**Figure 3:**
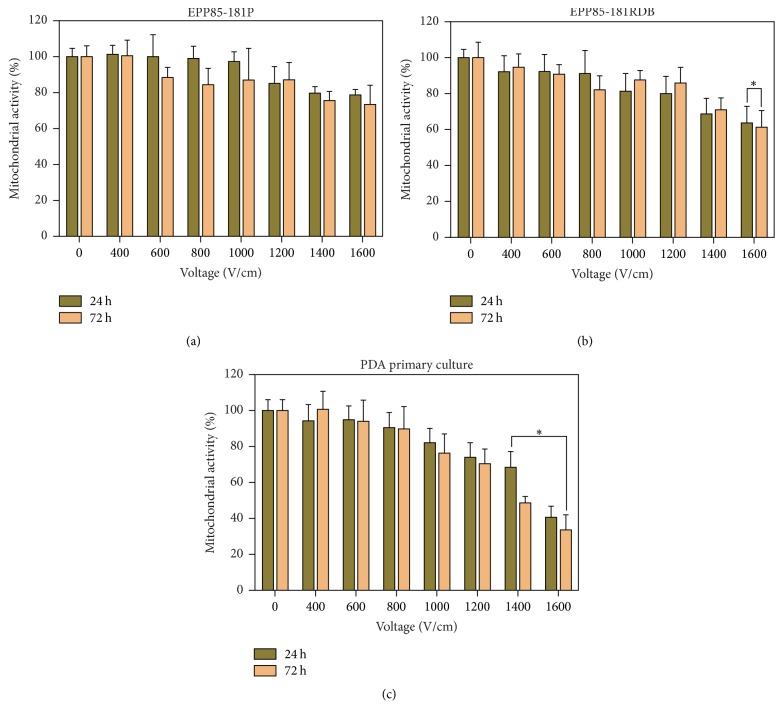
The results of MTT assay following the application of electric pulses of electric field strength at a range of 0–1600 V/cm on established cell lines of PDA sensitive (EPP85-181P; (a)) and resistant to daunorubicin (EPP85-181RDB; (b)) and on the primary cell culture from PDA pulmonary metastasis (c); after 24 and 72 h of incubation; ^*∗*^statistically significant differences between cells exposed to EP and nontreated control (*p* ≤ 0.05).

There was no significant impact of low electric field strength on the cells' viability, regardless of the origin of the cell line (Figure 3). The slight decrease in cells viability was observed following the application of electric field strength of 1000 V/cm. However, only after 1400 V/cm, the cell proliferation was disturbed and for the lower parameters the initial effect of the drop-in viability seems to be averted.

**Figure 4:**
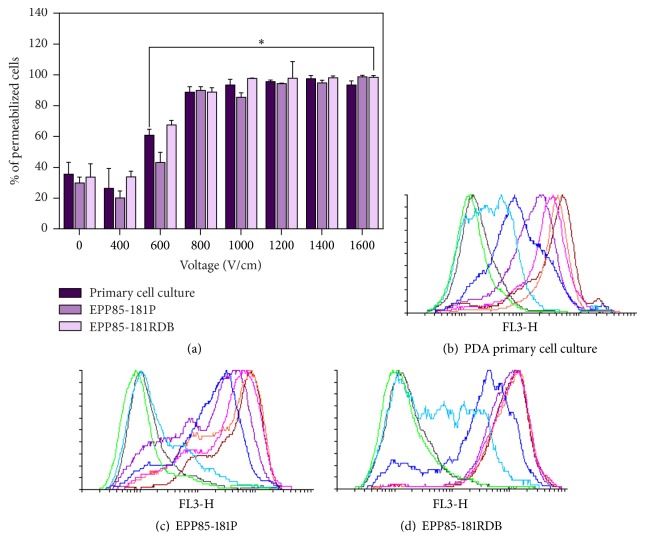
The percentage of permeabilized cells (a) and the propidium iodide fluorescence measured right after the electroporation with 10 *μ*M concentration of propidium iodide on three cell lines of PDA: primary cell culture of PDA metastatic cells from lung (b); EPP85-181P (sensitive to daunorubicin (c)) and EPP85-181RDB (resistant to daunorubicin (d)). The colors are assigned to the electric field strength: grey, 0 V/cm; green, 400 V/cm; blue, 600 V/cm; navy blue, 800 V/cm; violet, 1000 V/cm; pink, 1200 V/cm; orange, 1400 V/cm; red, 1600 V/cm; ^*∗*^statistically significant differences between cells exposed to EP and nontreated control (*p* ≤ 0.05).

In the course of study, we also performed electroporation with propidium iodide on the primary and established cell lines (Figure 4). The parameters of electroporation ranging from 800 to 1600 V/cm resulted in similar percentage of permeabilized cells in established cell lines as well as the primary cell culture (Figure 4(a)). However, lower parameters of 400 V/cm and 600 V/cm were more affecting the permeabilization in the primary cells and daunorubicin-resistant cell line EPP85-181 RDB than in the daunorubicin-sensitive cells. Interestingly, the uptake of the propidium iodide was the highest in P-gp overexpressing cells EPP85181RDB (Figure 4(d)), while the lowest fluorescence was detected in cells from the primary culture of PDA lung metastasis (Figure 4(b)). Therefore, based on literature [12, 22] and our experimental data, three different values were chosen for subsequent experiments: 600, 800, or 1000 V/cm.

### 3.3. Electroporation with Selected Cisplatin Concentration

**Figure 5:**
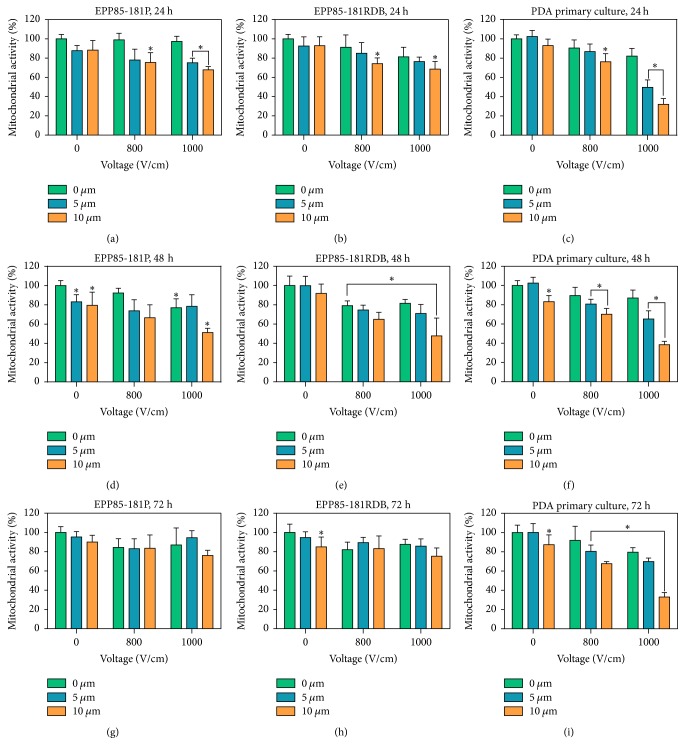
The results of MTT assay following CisEP with selected parameters on continuous PDA cell lines EPP85-181P (a, d, g) and EPP85-181RDB (b, e, h) and primary cell culture from PDA metastasis (c, f, i) after 24 (a, b, c), 48 (d, e, f), and 72 (g, h, i) hours of incubation; ^*∗*^statistically significant differences between groups: cis-treated or EP-treated versus nontreated control, ECT-treated versus nontreated, cis-treated, and EP-treated controls (*p* ≤ 0.05).

Enhanced cisplatin efficacy was observed in cells from PDAC pulmonary metastases when it was combined with electroporation (Figure 5). The most significant difference in cell viability was observed following 24 h incubation after ECT with 10 *μ*M cisplatin concentration and electric field strength of 1000 V/cm, in case of ECT-treated cells compared to the nontreated, cis-treated, and EP-treated control (*p* ≤ 0.05). For the primary cells following 24, 48, and 72 hours of incubation the viability was decreased by nearly 68, 61, and 67%, respectively, after electroporation with 10 *μ*M cisplatin, with respect to the nontreated control (Figures 5(c), 5(f), and 5(i)). However, for the lower concentration of cisplatin, the initially disturbed viability increased with the further time of incubation.

Surprisingly, the viability of cells after electroporation with cisplatin is not mirroring the sensitivity of primary and established cell lines to cisplatin measured via MTT assay. While cells from the primary cell culture showed the weakest response to drug itself, the cytotoxic effect of cisplatin when combined with electric pulses was significantly larger. In case of both established cell lines, the sensitive and resistant one to daunorubicin, the increased efficacy of cisplatin treatment was visible only 24 and 48 hours after procedure, while differences after 72 hours of incubation were minor and not statistically significant. In general, the drug-EP combination was equally effective for both established cell lines with a slight advantage for the daunorubicin-resistant cell line EPP85-181RDB in which the viability was decreased to 48% after 48 hours (Figure 5(e)), whereas in the daunorubicin-sensitive cell line it amounted to 51% of the control (Figure 5(d)).

The results of trypan blue staining suggest that 10 *μ*M can influence survival of cells from the primary cell culture, notwithstanding the short exposition to the drug (Table 2). However, drop in these cells' viability following ECT and 24 hours of incubation with a medium deprived of the drug indicates that there is an additive effect of EP and drug combination (Figure 5(c)).

### 3.4. The Evaluation of Superoxide Dismutase and Caspase 3 Immunoreactivity

The experiment was performed in order to verify the influence of the most efficient EP parameter (1000 V/cm) and a parameter resulting in approximately 60% permeabilization (600 V/cm) on the cellular stress occurrence. Although almost the entire subpopulation of PDA cells from the primary cell culture showed the expression of mitochondrial superoxide dismutase regardless of the experiment conditions, the immunoreaction was stronger after combining the application of cisplatin with electroporation (Table 3). Remarkably, it was observed only following the application of 1000 V/cm. For the lower parameter of electroporation there was no additive effect of cisplatin and electroporation, which can be explained by the insufficient permeabilization. For caspase 3, the increase of protein expression was detected after electroporation with both 600 and 1000 V/cm. However, the difference (precisely the strength of the reaction) was more significant for the higher parameter of the electric field strength. These results provide an evidence that electric pulses at a range of 1000 V/cm enhance the transport and finally anticancer effect of cisplatin, which may be a signal for oxidative stress or apoptosis. The lower parameter of electroporation also may generate a cellular response but via the action of electric field or drug rather than by enhanced transportation.

**Figure 6:**
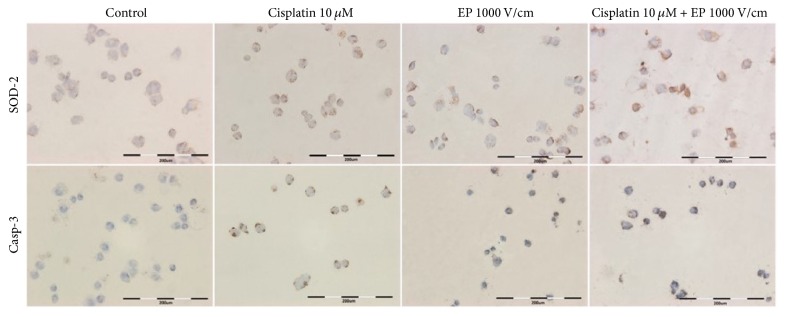
The disturbances of the morphology and the immunocytochemical reaction with SOD-2 and Casp-3 in the primary cell culture from pancreatic cancer pulmonary metastases following the CisEP experiment.

Predictably, disturbed morphology was observed after the application of electroporation alone as well as combined with cisplatin (Figure 6). While the cells' condition after the treatment with 10 *μ*M cisplatin for short time was comparable to the nontreated control. The cellular debris and shrinkage or contraction of the cytoplasm were visible following electroporation and ECT* in vitro*. Furthermore, the increase in the immunoreactivity of both oxidative stress markers after the combination of 1000 V with 10 *μ*M cisplatin indicates that the combination of these parameters was the most stressful for pancreatic cancer cells in the primary cell culture and thus the most effective as a therapeutic protocol in simulated, model conditions.

## 4. Discussion

Research conducted by Jaroszeski et al. (2000) revealed that the electric field strength of 900 V/cm decreases cells viability to 90% in the pancreatic cancer cell line Capan-2, while the same viability was observed after application of 750 V/cm in the Lewis lung carcinoma cell line after 48 hours of electroporation. In the same study, the increased uptake of bleomycin was confirmed after the application of electroporation with 900 V/cm in Capan-2 cells [12]. Although not as effective as with bleomycin, EP-enhanced drug transport was also observed in PDA cell lines when bleomycin was replaced with cisplatin. A study conducted on Capan-2 showed that electroporation can decrease cisplatin IC50 to 60 *μ*M [12] whereas similar cytotoxic effect was obtained after 24 hours of incubation on primary cells using only 5 *μ*M concentration of cisplatin. In the experiment conducted by Girelli group (2015) similar effect was observed on PANC1 cell line where cisplatin IC50 was decreased from 23 *μ*M to 8 *μ*M following the application of electric pulses. The response to the exposure to the electric field strength of 1000 V/cm was stronger for PANC1 than MiaPaCa2 cell line which was mirrored by a reduction of IC50 by factors 2.8 and 1.3, respectively [22]. In our study, the pancreatic cancer cells grown in primary cell culture are shown to be similar to the Capan-2 cell line sensitivity to electroporation; cells' viability was decreased by 9.45% for 800 V/cm and 17.81% for 1000 V/cm 24 hours after EP and by 10.19% for 800 V/cm and 23.68% for 1000 V/cm after 72 hours of incubation (Figure 3(c)).

Cisplatin contributes to the formation of various reactive oxygen species (ROS), which was demonstrated in a number of studies [23–26]. SOD2 is a mitochondrial manganese enzyme, which is the main scavenger of superoxide anions produced during the mitochondrial oxidative phosphorylation and therefore is present in cells and even not subjected to the experiment. There was described that many human cancer cells reveal low levels of MnSOD proteins and enzymatic activity, whereas some cancer cells possess high levels of MnSOD expression and activity [27]. In our study, the differences in SOD-2 expression between EP and drug alone or combined were slight. Interestingly, the oxidation of membrane components enhances the susceptibility of the membrane to the action of electropermeabilization [28]. Therefore, a longer incubation with cisplatin prior to electroporation or preincubation with low-toxic compound directed distinctly to the induction of oxidative stress in cell membranes could potentially reinforce the effect of ROS generation following the combination of cisplatin and electric pulses of low electric field strength.

In our experiment, we observed increased expression of apoptotic marker caspase 3 after electroporation with cisplatin. A similar reaction was demonstrated in the human bladder cancer cell line SW780 [29]; however, unlike this study, we noted increased caspase expression also after exposition to cisplatin alone. The increased apoptosis following electrochemotherapy* in vitro* seems promising as the induction of cell apoptosis can prevent the inflammatory responses* in vivo* [30].

In the case of bleomycin, the efficacy of electrochemotherapy was demonstrated on many different resistant cell lines [31–33]. In the study comparing sensitivity to the ECT, resistant cells showed a poorer response to bleomycin* in vitro* than* in vivo*; hence it has been suggested that the difference in sensitivity to bleomycin and ECT predominantly reflects the difference in intrinsic sensitivity of the cells [34]. In our research, cells from the primary cell culture showed a significantly higher response to electrochemotherapy with cisplatin when compared to the continuous cell lines EPP85-181P and EPP85-181RDB, notwithstanding the poorer sensitivity to drug alone. This result corresponds to the results obtained by Kulbacka et al. (2014) on different human adenocarcinoma cell lines with overexpression of P-glycoprotein and murine macrophage cells which revealed that electrochemotherapy with doxorubicin induces changes in P-gp expression and affects cellular ultrastructure; hence the most visible effect concerned resistant cell lines with P-gp overexpression [31]. A similar effect was obtained both* in vitr*o and* in vivo* by Meschini et al. (2012) [35]. However, despite the expression of P-glycoprotein in the vast majority of pancreatic cancer cells [36], the additive effect of EP and cisplatin obtained in our study cannot be attributed to P-glycoprotein inhibition, especially that the mechanisms of resistance to platinum-based antitumor agents are MDR1-independent [37]. On the other hand, some authors indicate that cisplatin sensitivity might be related to high overexpression of MUC4, NF-*κ*B, and MDR, for example in Capan-2 cell [38, 39]. This could translate into the efficiency of the drug transport via the electroporation.

The study with cisplatin conducted by Cemazar et al. (2001)* in vitro* revealed that cells sensitive to cisplatin are equally sensitive to ECT with cisplatin as their cisplatin-sensitive counterparts, while* in vivo* experiment demonstrated that ECT is more effective for parental tumors [40]. According to the authors, results obtained* in vitro* might indicate that the membrane restriction of cisplatin uptake can be a predominant mechanism of resistance. Our study was performed on cells from the metastasis (not derived from primary tumor) and the results of MTT assay following incubation with cisplatin indicate that resistance to cisplatin may also be the reason of weak cytotoxic effect in our research. The mechanisms distinguishing the response exhibited by cells from primary tumors and metastasis of PDA remain unrevealed. Certainly, the response can be different as during the process of metastasis cells go through detachment, migration, invasion, and adhesion. These steps are interrelated and affected by multibiochemical events and parameters [41]. Moreover, it has been shown that primary and metastatic cells may differ in stiffness, adhesion, cells shape, actin organization, and surface roughness in order to better fulfill their role in cancer growth and invasion [42].

Up to now, there is no available data regarding the different primary cancer cell lines exposed to electrochemotherapeutic protocol. Frandsen et al. (2016) investigated the difference in membrane repair capacity of the normal primary cells and the malignant melanoma and observed that the membrane repair was more effective for the primary cells. The authors suggested that this may explain why other therapies permeabilizing the plasma membrane are more effective in malignant cells compared to normal cells in cancer treatment [43].

## 5. Conclusions

Pancreatic cancer is still a serious challenge for clinicians. The results presented here indicate that electroporation can be effectively applied even when cells initially do not respond to cisplatin. We have demonstrated a selective effect on cell survival after electroporation with cisplatin* in vitro* on the human primary cells derived from the pulmonary metastasis of pancreatic ductal adenocarcinoma. Our studies confirm that electroporation enhances drug cytotoxicity and thereby may be involved in apoptosis and oxidative stress generation. Importantly, we noticed that while some parameters reflected the results obtained in established cell lines, some other crucial factors such as drug concentration influenced cells derived from tumor in a completely different manner. Therefore, it seems relevant to additionally utilize primary cultures as models a bit closer to the actual tissue, especially regarding the chemo- end electrosensitivity testing.

## Figures and Tables

**Table 1:** Immunoreactivity of pancreatic adenocarcinoma cells from primary cell culture, passage 5 (P5), and passage 20 (P20), with antibodies against TTF-1, CK-7, CK-20, and GP2.

*Antibody*	*Immunocytochemical reaction*	
	P5	P20
TTF-1	*Negative*	*Negative*
CK 7	*Positive*	*Positive*
CK 20	*Positive*	*Positive*
GP2	*Positive*	*Positive*

**Table 2:** The results of trypan blue staining of pancreatic cancer cells from primary cell culture 30 min after electroporation with electric field strength of 1000 V/cm and 10 *µ*M cisplatin.

Treatment	% of viable cells	SD
Control	90	±4.9
Cisplatin	74	±9.5
EP	86	±7.2
CisEP	60	±9.5

**Table 3:** The results of immunocytochemical staining for the reactivity with mitochondrial superoxide dismutase (SOD-2) and the apoptotic marker caspase 3 (Casp-3) on human primary cell culture derived from PDA pulmonary metastases.

Electric field strength	Cisplatin concentration [*µ*M]	SOD-2		Casp-3	
		Staining intensity	% of stained cells	Staining intensity	% of stained cells
0 V/cm	0	+	100	−/+	22
	5	+/++	100	+	78
	10	+/++	100	+/++	97

600 V/cm	0	+/++	100	+	96
	5	+/++	100	++	98
	10	+/++	100	++/+++	97

1000 V/cm	0	++	100	+	90
	5	++/+++	100	++/+++	100
	10	+++	100	++/+++	100
